# Silicosis Diagnosed Using Transbronchial Lung Biopsy: The Pivotal Role of Occupational History

**DOI:** 10.1002/ccr3.71759

**Published:** 2026-01-04

**Authors:** Boon Hau Ng, Nor Safiqah Sharil, Hsueh Jing Low, Nik Nuratiqah Nik Abeed, Badrul Iskandar Abdul Wahab, Rose Azzlinda Osman, Andrea Yu‐Lin Ban

**Affiliations:** ^1^ Respiratory Unit, Department of Medicine, Faculty of Medicine Universiti Kebangsaan Malaysia, Hospital Canselor Tuanku Muhriz Kuala Lumpur Malaysia; ^2^ Internal Medical Unit, Faculty of Medicine and Health Science Universiti Sains Islam Malaysia Nilai Negeri Sembilan Malaysia; ^3^ Department of Anaesthesiology and Critical Care, Faculty of Medicine Universiti Kebangsaan Malaysia, Hospital Canselor Tuanku Muhriz Kuala Lumpur Malaysia; ^4^ Department of Diagnostic Laboratory Services, Faculty of Medicine Universiti Kebangsaan Malaysia, Hospital Canselor Tuanku Muhriz Kuala Lumpur Malaysia

**Keywords:** chest radiograph, occupational history, reticulonodular opacities, silicosis, transbronchial lung biopsy

## Abstract

Pulmonary silicosis, a preventable occupational lung disease caused by chronic inhalation of crystalline silica dust in industries like construction, mining, and stone masonry, remains underdiagnosed in TB‐endemic regions due to overlapping clinical and radiological features. A 63‐year‐old man presented with upper gastrointestinal bleeding, but a silent lung abnormality stole the clinical spotlight. In a TB‐endemic setting, incidental reticulonodular opacities triggered a full tuberculosis work‐up, even in the absence of cough or respiratory symptoms. Yet it was only after a delayed occupational history revealed three decades of unprotected mosaic tiling that silicosis was considered. High‐resolution CT and transbronchial lung biopsy later confirmed the diagnosis, but not before the patient's course was complicated by obstructive uropathy and sepsis. This case highlights the diagnostic pitfalls of radiologic overlap between silicosis and tuberculosis, as well as the crucial role of occupational exposure history in reaching the correct diagnosis.

## Key Clinical Message

In TB‐endemic regions, incidental lung findings may be misleading. This case highlights the diagnostic challenge of distinguishing silicosis from tuberculosis and emphasizes that a thorough occupational exposure history, coupled with transbronchial lung biopsy, is essential for accurate diagnosis, especially when radiologic features overlap and respiratory symptoms are absent.

## 1 Introduction

Pulmonary silicosis is an occupational lung disease resulting from the chronic inhalation of crystalline silica dust, typically affecting individuals in industries such as construction, mining, and stone masonry. Despite being a preventable condition, silicosis remains underdiagnosed, particularly in regions where TB is endemic, due to overlapping clinical and radiological features [1]. Patients with silicosis may present with minimal or no respiratory symptoms, and imaging findings such as reticulonodular infiltrates or upper lobe predominance often mimic pulmonary tuberculosis [2]. In such contexts, there is a high risk of misdiagnosis, which may lead to inappropriate treatment and delayed definitive care.

We report an elderly man presenting with upper gastrointestinal bleeding and incidental bilateral pulmonary opacities, initially presumed to be tuberculosis, but later diagnosed as silicosis after a delayed occupational history revealed chronic silica exposure and histopathology confirmed the diagnosis.

## 2 Case History/Examination

A 63‐year‐old man was admitted with a 6‐day history of melaena, accompanied by dizziness and lethargy. He had been self‐medicating intermittently with over‐the‐counter analgesics. On examination, he appeared pale, with investigations revealing severe anemia (Hb 4 g/dL) (normal range: 13.5–17.5 g/dL) and hypoalbuminemia. Urgent esophagogastroduodenoscopy showed a prepyloric Forrest 2B ulcer with an adherent clot and pangastritis, for which adrenaline injection and haemoclip application were performed. He received a total of five units of blood and was started on a proton pump inhibitor infusion.

## 3 Differential Diagnosis, Investigations, and Treatment

**FIGURE 1:**
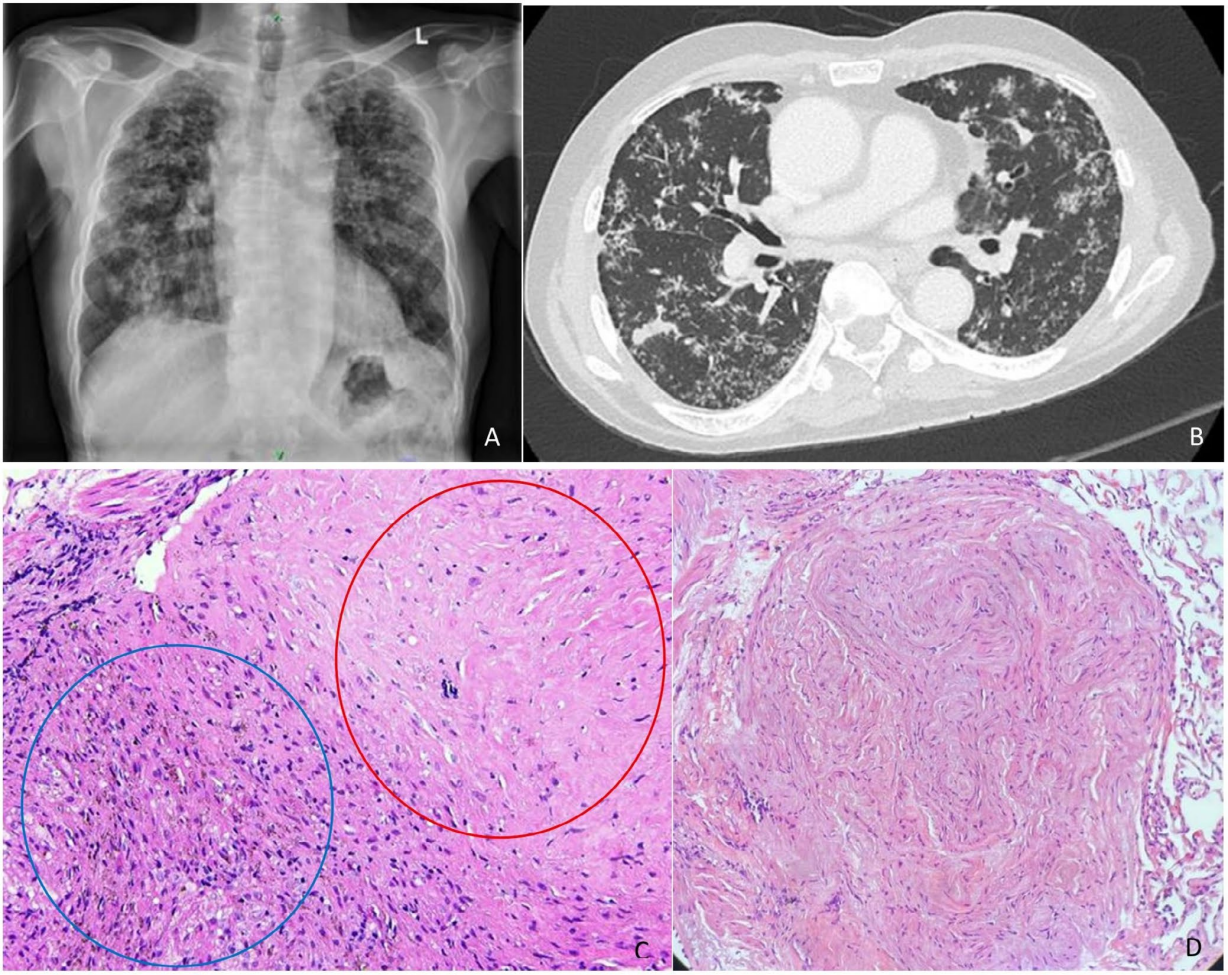
(A) Chest radiograph showed bilateral reticulonodular opacities. (B) Contrast enhanced computed tomography of the thorax showed centrilobular and tree‐in‐buds lung nodules scattered in both lung fields. (C) Histopathological examination (HPE) of the transbronchial lung biopsy showed an acellular nodule consisting of hyaline collagen deposition (red circle), and it is surrounded by aggregates of foamy histiocytes and pigment‐laden histiocytes (blue circle) (H&E stain, ×400 magnification). (D) HPE revealed a fibrotic nodule (H&E stain, ×200 magnification).

During admission, the patient reported unintentional weight loss of approximately five kilograms over the past 3 years, which raised clinical suspicion for pulmonary tuberculosis (PTB), despite the absence of cough or other respiratory symptoms. A chest radiograph obtained on the day of admission demonstrated bilateral diffuse reticulonodular opacities, predominantly in the upper and mid lung zones, without evidence of cavitation, consolidation, or pleural effusion (Figure 1A). Although non‐specific, these radiographic findings were concerning for PTB. A full tuberculous (TB) work‐up was initiated. Within 48 h, the respiratory team was consulted. Despite clear lung fields on auscultation and stable vital signs, the patient was presumed to have PTB and was placed under airborne isolation. Plans were made for induced sputum sampling for acid‐fast bacilli (AFB) and diagnostic bronchoscopy. Serial sputum samples were negative for acid‐fast bacilli (AFB) and *Mycobacterium tuberculosis* by GeneXpert and mycobacterial culture. The interferon‐gamma release assay was also negative.

By the end of the first week, a more detailed review by a respiratory physician uncovered a significant occupational history: the patient had worked for over 30 years as a mosaic tiler without the use of respiratory protective equipment. He also had a 30‐pack‐year smoking history. This key information shifted the diagnostic consideration from TB to pulmonary silicosis. A high‐resolution CT (HRCT) of the thorax was subsequently performed, revealing diffuse centrilobular nodules, tree‐in‐bud opacities, and patchy consolidation (Figure 1B), findings that were non‐specific for both silicosis and active PTB, which require further diagnostic clarification.

Before tissue confirmation could be obtained, the patient developed obstructive uropathy due to right distal ureteric calculi, leading to acute kidney injury. Plans for bronchoscopy with transbronchial lung biopsy (TBLB) were deferred while he underwent nephrostomy insertion and stabilization. Two months after the initial admission, he presented again with sepsis and fast atrial fibrillation secondary to urosepsis from ESBL‐producing *Klebsiella pneumoniae* , which was managed with targeted antibiotics and supportive care.

Bronchoscopy and TBLB were eventually performed approximately 13 weeks after the initial presentation, following stabilization from unrelated urological complications. Histopathological examination of the biopsy specimens revealed fibrotic nodules characteristic of silicotic nodules, accompanied by chronic non‐caseating granulomatous inflammation (Figure 1C,D). Investigations for tuberculosis, including the bronchoalveolar lavage (BAL) for acid‐fast bacilli, GeneXpert, and mycobacterial culture, were negative. Pulmonary tuberculosis was excluded based on a negative BAL result, while histopathological examination revealed silicotic nodules without caseating granulomas. Together with the patient's significant history of occupational silica exposure, these findings strongly supported the diagnosis of silicosis.

## 4 Discussion

This case illustrates the diagnostic complexity of distinguishing silicosis from pulmonary PTB, particularly in TB‐endemic regions where systemic symptoms such as weight loss may easily prompt a presumptive TB diagnosis. Our patient presented with upper gastrointestinal bleeding, but incidental chest radiography revealed bilateral upper‐ and mid‐zone reticulonodular opacities. Despite the absence of respiratory symptoms, the imaging raised suspicion of PTB, triggering airborne isolation and an expedited TB work‐up, standard practice in high‐prevalence settings [3].

The differential diagnosis for diffuse pulmonary opacities is broad and includes TB, silicosis, sarcoidosis, metastatic disease, hypersensitivity pneumonitis, and other pneumoconioses. Diagnostic tests such as the Mantoux test have limited sensitivity (approximately 70%–90%) and specificity, particularly in BCG‐vaccinated populations [4]. BAL for AFB offers sensitivity ranging from 50% to 80% in smear‐positive cases, but falls significantly in smear‐negative or disseminated TB [5]. The sensitivity of the BAL MTB GeneXpert assay has been reported to range between 80% and 90%, and in some studies, even higher [6].

It was only after a delayed, thorough occupational history was obtained more than a week after admission that the possibility of silicosis was considered. The patient had worked for over 30 years as a mosaic tiler without respiratory protection, a job associated with chronic exposure to silica dust. A high‐quality occupational history includes job titles, specific tasks, duration of employment, materials used, workplace controls (e.g., ventilation), use of personal protective equipment, and similar exposures among co‐workers [7].

HRCT of the thorax showed diffuse centrilobular nodules, perilymphatic distribution, “tree‐in‐bud” opacities, and subpleural fibrosis, hallmark features of silicosis, particularly when upper‐lobe predominant [8, 9]. However, these radiologic findings can also overlap with those of TB and require histological confirmation in diagnostically ambiguous cases. TBLB, though not routinely needed, was performed to exclude concurrent TB or malignancy [10]. Histopathology revealed fibrotic silicotic nodules and non‐caseating granulomatous inflammation, supporting a diagnosis of silicosis [11]. It is important to note that TBLB has limitations in sensitivity for silicosis, and while surgical lung biopsy (e.g., via VATS) may offer a higher diagnostic yield, it carries a greater procedural risk.

HRCT of the thorax is a valuable tool in diagnosing silicosis, particularly in patients with a history of silica exposure. However, in our patient, the HRCT findings were non‐specific and warrant a lung biopsy to exclude other differential diagnoses. Lung biopsy provides direct histopathological confirmation of silicotic nodules and helps rule out alternative conditions such as granulomatous infection or malignancy. Nevertheless, it carries inherent disadvantages, including its invasive nature, procedural risks (e.g., bleeding, pneumothorax), and the possibility of sampling error in heterogeneous disease.

The classic histopathological feature of silicosis is the silicotic nodule. Microscopically, these nodules exhibit two distinct zones: a central and a peripheral region. The central zone is composed of concentrically arranged collagen fibers, while the peripheral zone is less organized and contains fibroblasts, lymphocytes, and dust‐laden macrophages [12, 13]. Under polarized light microscopy, weakly birefringent polyhedral particles and brightly birefringent needle‐shaped silicate crystals can be observed [12, 13]. In advanced stages, multiple nodules may merge, leading to the formation of progressive massive fibrosis.

Complicating this patient's diagnostic journey were unrelated but significant comorbidities. He developed obstructive uropathy due to bilateral nephrolithiasis and ureteric calculi, leading to acute kidney injury and the need for nephrostomy. This delayed bronchoscopy contributed to a prolonged hospital stay, which was further complicated by urosepsis and atrial fibrillation.

## 5 Conclusion

This case highlights the importance of a comprehensive occupational history in evaluating patients with unexplained pulmonary opacities, even when respiratory symptoms are absent. In TB‐endemic regions, anchoring bias toward tuberculosis can overshadow alternative diagnoses such as silicosis, especially when radiologic features overlap. Early consideration of occupational lung disease and structured history‐taking, complemented by minimally invasive lung biopsy, can prevent misdiagnosis and guide appropriate management.

## Author Contributions

**Boon Hau Ng:** conceptualization, data curation, writing – original draft, writing – review and editing. **Nor Safiqah Sharil:** data curation, supervision, writing – review and editing. **Nik Nuratiqah Nik Abeed:** data curation, supervision, writing – review and editing. **Hsueh Jing Low:** conceptualization, data curation, writing – original draft, writing – review and editing. **Badrul Iskandar Abdul Wahab:** investigation. **Rose Azzlinda Osman:** data curation, supervision, writing – review and editing. **Andrea Yu‐Lin Ban:** data curation, supervision, writing – review and editing.

## Funding

The authors have nothing to report.

## Ethics Statement

The current study did not require ethical approval, as it was conducted in accordance with local ethical guidelines. The study was conducted in accordance with the Helsinki Declaration, and informed consent was obtained from patients to discuss or publish details of their disease, images, and treatment course.

## Consent

Written informed consent for publication was obtained from the patient.

## Conflicts of Interest

The authors declare no conflicts of interest.

## Data Availability

The data that support the findings of this study are available from the corresponding author upon reasonable request.
